# Entropy Analysis of the Peristaltic Flow of Hybrid Nanofluid Inside an Elliptic Duct with Sinusoidally Advancing Boundaries

**DOI:** 10.3390/e23060732

**Published:** 2021-06-09

**Authors:** Luthais B. McCash, Salman Akhtar, Sohail Nadeem, Salman Saleem

**Affiliations:** 1 School of Mathematics & Actuarial Science, University of Leicester, Leicester LE1 7RH, UK; lm460@leicester.ac.uk; 2 Department of Mathematics, Quaid-i-Azam University 45320, Islamabad 44000, Pakistan; sakhtar@math.qau.edu.pk; 3 Department of Mathematics, College of Science, King Khalid University, Abha 61413, Saudi Arabia; saakhtar@kku.edu.sa

**Keywords:** peristalsis, elliptic duct, hybrid nanofluid, entropy analysis

## Abstract

Peristaltic flow of hybrid nanofluid inside a duct having sinusoidally advancing boundaries and elliptic cross-section is mathematically investigated. The notable irreversibility effects are also examined in this mathematical research by considering a descriptive entropy analysis. In addition, this work provides a comparison analysis for two distinct nanofluid models: a hybrid model (Cu-Ag/water) and a phase flow model (Cu/water). A comprehensive graphical description is also provided to interpret the physical aspects of this mathematical analysis.

## 1. Introduction

Many recent functional devices operate on the key principle of peristalsis. These are useful in industry, engineering and medical fields, etc., for food, corrosive liquids, blood, and chemical transportation [1]. The flow within a tube due to the sinusoidal wall motion that propels the fluid along the axial length of this tube was mathematically evaluated by Barton and Raynor [2]. Siddiqui and Schwarz [3] had conveyed the analytical analysis of non-Newtonian flow within a tube due to the sinusoidal wall motion of this tube. Our main focus in this investigation is to highlight the physical aspects and dynamics of flow within a duct that has an elliptic cross-section. Saleem et al. [4] recently provided the first research work that evaluates the heated flow within a duct that has an elliptic cross section and fluctuating sinusoidal walls. Further, Nadeem et al. [5] provided a comprehensive analysis with a thorough, streamlined evaluation for heated non-Newtonian flow inside a duct with an elliptic cross-section.

Nanofluids are used in the base fluid for a required rate of thermal conductivity that helps in achieving the target of a descriptive heat transfer mechanism. Sometimes, we use more than one nanofluid in the base fluid. These are then called hybrid nanofluids. Akbar and Nadeem [6] provided an analysis of nanofluid flow within a tube that has sinusoidally deforming walls. Tripathi and Beg [7] mathematically evaluated the peristaltic flow of nanofluid and also targeted its prime drug delivery applications in their study. Khan et al. [8] modelled the peristaltic flow phenomenon by considering the various types of nanofluids inside an asymmetric channel. The flow of nanofluids with applications of a peristalsis mechanism within a hybrid model was evaluated by Awais et al. [9]. Bibi and Xu [10] also took into account the chemical reaction activity occurring during the peristaltic flow with a hybrid model of nanofluids. Further to this, recent literature that interprets the peristaltic flow with nano-fluidic applications has been conveyed [11,12,13,14,15,16,17,18,19].

Entropy analysis has also been mathematically studied by many recent researchers to interpret the irreversibility effects and disorder of the whole system. Akbar [20] provided an interpretation of entropy generation for peristaltic flow problems. Akbar et al. [21] also evaluated the nanofluid flow with entropy analysis for peristaltic flow problems. Hayat et al. [22] recently conveyed a mathematical analysis of hybrid nanofluids flow inside a sinusoidally deforming channel with entropy evaluation. The three-dimensional flow analysis inside a cylindrical cavity with nanofluids and entropy evaluation was modelled by Riaz et al. [23]. Additional recent studies on this topic are presented in [24,25,26,27,28,29].

The in-depth and descriptive literature analysis reveals that the peristaltic flow of hybrid nanofluid inside an elliptic duct has not yet been mathematically investigated. Furthermore, we have incorporated the irreversibility effects by considering entropy analysis in the present study. Momentum and energy equations are solved exactly by utilizing a polynomial solution approach. Finally, we have evaluated a descriptive graphical analysis that not only highlights the physical aspects of this mathematical study but also verifies the mathematical computations.

## 2. Mathematical Model

**Figure 1:**
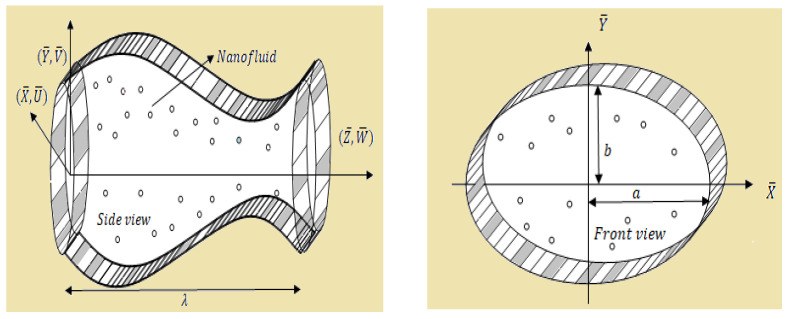
Geometry of the problem.

The geometrical model for this flow problem is presented by Figure 1.

The sinusoidal motion of this duct’s boundary is mathematically considered by utilizing the following equations:

(1) $$\bar{a}\left(\bar{Z},\bar{t}\right)=a_{0}+dsin\left(\frac{2\pi }{\lambda }\left(\bar{Z}-c\bar{t}\right)\right)\;\bar{b}\left(\bar{Z},\bar{t}\right)=b_{0}+dsin\left(\frac{2\pi }{\lambda }\left(\bar{Z}-c\bar{t}\right)\right)$$

The primary mathematical equations that elucidate the hybrid nanofluid flow inside this elliptic duct are unfolded as follows:

(2) $$\frac{\partial \bar{U}}{\partial \bar{X}}+\frac{\partial \bar{V}}{\partial \bar{Y}}+\frac{\partial \bar{W}}{\partial \bar{Z}}=0$$

(3) $$\rho_{hnf}\left(\frac{\partial \bar{U}}{\partial \bar{t}}+\bar{U}\frac{\partial \bar{U}}{\partial \bar{X}}+\bar{V}\frac{\partial \bar{U}}{\partial \bar{Y}}+\bar{W}\frac{\partial \bar{U}}{\partial \bar{Z}}\right)=-\frac{\partial \bar{P}}{\partial \bar{X}}+\mu_{hnf}\left(\frac{\partial^{2}\bar{U}}{\partial {\bar{X}}^{2}}+\frac{\partial^{2}\bar{U}}{\partial {\bar{Y}}^{2}}+\frac{\partial^{2}\bar{U}}{\partial {\bar{Z}}^{2}}\right)$$

(4) $$\rho_{hnf}\left(\frac{\partial \bar{V}}{\partial \bar{t}}+\bar{U}\frac{\partial \bar{V}}{\partial \bar{X}}+\bar{V}\frac{\partial \bar{V}}{\partial \bar{Y}}+\bar{W}\frac{\partial \bar{V}}{\partial \bar{Z}}\right)=-\frac{\partial \bar{P}}{\partial \bar{Y}}+\mu_{hnf}\left(\frac{\partial^{2}\bar{V}}{\partial {\bar{X}}^{2}}+\frac{\partial^{2}\bar{V}}{\partial {\bar{Y}}^{2}}+\frac{\partial^{2}\bar{V}}{\partial {\bar{Z}}^{2}}\right)$$

(5) $$\rho_{hnf}\left(\frac{\partial \bar{W}}{\partial \bar{t}}+\bar{U}\frac{\partial \bar{W}}{\partial \bar{X}}+\bar{V}\frac{\partial \bar{W}}{\partial \bar{Y}}+\bar{W}\frac{\partial \bar{W}}{\partial \bar{Z}}\right)=-\frac{\partial \bar{P}}{\partial \bar{Z}}+\mu_{hnf}\left(\frac{\partial^{2}\bar{W}}{\partial {\bar{X}}^{2}}+\frac{\partial^{2}\bar{W}}{\partial {\bar{Y}}^{2}}+\frac{\partial^{2}\bar{W}}{\partial {\bar{Z}}^{2}}\right)$$

(6) $$\begin{array}{ll} {\left(\rho C_{p}\right)}_{hnf}(\frac{\partial \bar{T}}{\partial \bar{t}} & +\bar{U}\frac{\partial \bar{T}}{\partial \bar{X}}+\bar{V}\frac{\partial \bar{T}}{\partial \bar{Y}}+\bar{W}\frac{\partial \bar{T}}{\partial \bar{Z}})\\ \; & =k_{hnf}\left(\frac{\partial^{2}\bar{T}}{\partial {\bar{X}}^{2}}+\frac{\partial^{2}\bar{T}}{\partial {\bar{Y}}^{2}}+\frac{\partial^{2}\bar{T}}{\partial {\bar{Z}}^{2}}\right)\\ \; & +\mu_{hnf}[2\left\{{\left(\frac{\partial \bar{U}}{\partial \bar{X}}\right)}^{2}+{\left(\frac{\partial \bar{V}}{\partial \bar{Y}}\right)}^{2}+{\left(\frac{\partial \bar{W}}{\partial \bar{Z}}\right)}^{2}\right\}+{\left(\frac{\partial \bar{U}}{\partial \bar{Y}}+\frac{\partial \bar{V}}{\partial \bar{X}}\right)}^{2}+{\left(\frac{\partial \bar{V}}{\partial \bar{Z}}+\frac{\partial \bar{W}}{\partial \bar{Y}}\right)}^{2}\\ \; & +{\left(\frac{\partial \bar{W}}{\partial \bar{X}}+\frac{\partial \bar{U}}{\partial \bar{Z}}\right)}^{2}] \end{array}$$

The suitable boundary conditions are provided in dimensional form as follows:

(7) $$\bar{W}=0,\;\bar{T}={\bar{T}}_{w},\;\mathrm{for}\;\frac{{\bar{x}}^{2}}{{\bar{a}}^{2}}+\frac{{\bar{y}}^{2}}{{\bar{b}}^{2}}=1$$

The two relevant reference frames are connected through these mathematical equations.

(8) $$\bar{x}=\bar{X},\;\bar{y}=\bar{Y},\;\bar{z}=\bar{Z}-c\bar{t},\;\bar{p}=\bar{P},\;\bar{u}=\bar{U},\;\bar{v}=\bar{V},\;\bar{w}=\bar{W}-c,$$

The relevant non-dimensional parameters that are utilized in this problem to obtain the simplified dimensionless form of mathematical equations are provided as:

(9) $$\begin{array}{c} x=\frac{\bar{x}}{D_{h}},\;y=\frac{\bar{y}}{D_{h}},\;z=\frac{\bar{z}}{\lambda },\;t=\frac{c\bar{t}}{\lambda },\;w=\frac{\bar{w}}{c},\;p=\frac{D_{h}^{2}\bar{p}}{\mu_{f}\lambda c},\;\theta =\frac{\bar{T}-{\bar{T}}_{w}}{{\bar{T}}_{b}-{\bar{T}}_{w}},\;\delta =\frac{b_{0}}{a_{0}},\;\phi =\frac{d}{b_{0}}\\ u=\frac{\lambda \bar{u}}{D_{h}c},\;v=\frac{\lambda \bar{v}}{D_{h}c},\;a=\frac{\bar{a}}{D_{h}},\;b=\frac{\bar{b}}{D_{h}},\;B_{r}=\frac{\mu_{f}c^{2}}{k_{f}\left({\bar{T}}_{b}-{\bar{T}}_{w}\right)}\\ \Omega =\frac{{\bar{T}}_{b}-{\bar{T}}_{w}}{{\bar{T}}_{w}},\;S=\frac{\bar{S}}{\frac{k_{f}{\left({\bar{T}}_{b}-{\bar{T}}_{w}\right)}^{2}}{D_{h}{}^{2}{\bar{T}}_{w}{}^{2}}} \end{array}$$

where $D_{h}$ denotes the hydraulic diameter, defined as:

(10) $$D_{h}=\frac{\pi b_{0}}{E\left(e\right)}$$

Moreover, E(e) is defined as [30] and $e=\sqrt{1-\delta^{2}}$.

(11) E(e)=∫0π21−e2sin2αdα

The final simplified and dimensionless form of governing mathematical equations is provided as

(12) $$\frac{\partial p}{\partial x}=0$$

(13) $$\frac{\partial p}{\partial y}=0$$

(14) $$\frac{dp}{dz}=\left(\frac{\mu_{hnf}}{\mu_{f}}\right)\left(\frac{\partial^{2}w}{\partial x^{2}}+\frac{\partial^{2}w}{\partial y^{2}}\right)$$

(15) $$\left(\frac{k_{hnf}}{k_{f}}\right)\left(\frac{\partial^{2}\theta }{\partial x^{2}}+\frac{\partial^{2}\theta }{\partial y^{2}}\right)+B_{r}\left(\frac{\mu_{hnf}}{\mu_{f}}\right)\left[{\left(\frac{\partial w}{\partial x}\right)}^{2}+{\left(\frac{\partial w}{\partial y}\right)}^{2}\right]=0$$

The non-dimensional form of boundary conditions is given as

(16) $$w=-1,\;\mathrm{for}\;\frac{x^{2}}{a^{2}}+\frac{y^{2}}{b^{2}}=1,$$

(17) $$\theta =0,\;\mathrm{for}\;\frac{x^{2}}{a^{2}}+\frac{y^{2}}{b^{2}}=1$$

and $a=\frac{E\left(e\right)}{\pi }\left[\frac{1}{\delta }+\phi sin\left(2\pi z\right)\right],$ and $b=\frac{E\left(e\right)}{\pi }\left[1+\phi sin\left(2\pi z\right)\right].$

Table 1 and Table 2 represents the numerical values and experimental formulas for thermo physical features of hybrid nanofluid respectively.

## 3. Entropy Analysis

The dimensional mathematical formulation for the entropy generation is written as [26]

(18) $$\bar{S}=\frac{k_{hnf}}{{\bar{T}}_{w}^{2}}\left[{\left(\frac{\partial \bar{T}}{\partial \bar{X}}\right)}^{2}+{\left(\frac{\partial \bar{T}}{\partial \bar{Y}}\right)}^{2}+{\left(\frac{\partial \bar{T}}{\partial \bar{Z}}\right)}^{2}\right]+\frac{\mu_{hnf}}{{\bar{T}}_{w}}\left[{\left(\frac{\partial \bar{W}}{\partial \bar{X}}\right)}^{2}+{\left(\frac{\partial \bar{W}}{\partial \bar{Y}}\right)}^{2}+{\left(\frac{\partial \bar{W}}{\partial \bar{Z}}\right)}^{2}\right]$$

The dimensionless and simplified mathematical form of entropy equation is given as follows:

(19) $$S=\left(\frac{k_{hnf}}{k_{f}}\right)\left[{\left(\frac{\partial \theta }{\partial x}\right)}^{2}+{\left(\frac{\partial \theta }{\partial y}\right)}^{2}\right]+\left(\frac{\mu_{hnf}}{\mu_{f}}\right)\frac{B_{r}}{\Omega }\left[{\left(\frac{\partial w}{\partial x}\right)}^{2}+{\left(\frac{\partial w}{\partial y}\right)}^{2}\right]$$

Moreover, the Bejan number, defined as the ratio of entropy produced due to conduction and total entropy, is given as

(20) $$B_{e}=\frac{S_{cond.}}{S_{cond.}+S_{visc.}}$$

After using the relevant values in Equation (20), we get

(21) $$B_{e}=\frac{\left(\frac{k_{hnf}}{k_{f}}\right)\left[{\left(\frac{\partial \theta }{\partial x}\right)}^{2}+{\left(\frac{\partial \theta }{\partial y}\right)}^{2}\right]}{\left(\frac{k_{hnf}}{k_{f}}\right)\left[{\left(\frac{\partial \theta }{\partial x}\right)}^{2}+{\left(\frac{\partial \theta }{\partial y}\right)}^{2}\right]+\left(\frac{\mu_{hnf}}{\mu_{f}}\right)\frac{B_{r}}{\Omega }\left[{\left(\frac{\partial w}{\partial x}\right)}^{2}+{\left(\frac{\partial w}{\partial y}\right)}^{2}\right]},$$

## 4. Exact Solution

Let

(22) $$w\left(x,y\right)=C_{1}x^{4}+C_{2}y^{4}+C_{3}x^{2}y^{2}+C_{4}x^{2}+C_{5}y^{2}+C_{6}$$

The value of w(x,y) given in Equation (22) is inserted in momentum Equation (14) and the coefficients of $x^{2},\;y^{2},\;x^{0},\;y^{0}$ are compared to get

(22a) $$12C_{1}+2C_{3}=0$$

(22b) $$2C_{3}+12C_{2}=0$$

(22c) 2C4+2C5=dpdzμhnfμf

Additionally, by using Equation (22) in the boundary condition for momentum equation provided in Equation (16) and comparing the coefficients of $x^{4},\;x^{2},\;x^{0}$, we have

(22d) $$C_{1}a^{4}+C_{2}b^{4}-C_{3}a^{2}b^{2}=0$$

(22e) $$-2C_{2}b^{4}+C_{3}a^{2}b^{2}+C_{4}a^{2}-C_{5}b^{2}=0$$

(22f) $$C_{2}b^{4}+C_{5}b^{2}+C_{6}=-1$$

The simultaneous solution of Equations (22a)–(22f) give the values of above constants as follows:

$$\begin{array}{c} C_{1}=0,\;C_{2}=0,\;C_{3}=0,\;C_{4}=\frac{b^{2}\frac{dp}{dz}}{2\left(a^{2}+b^{2}\right)\left(\frac{\mu_{hnf}}{\mu_{f}}\right)},\\ C_{5}=\frac{a^{2}\frac{dp}{dz}}{2\left(a^{2}+b^{2}\right)\left(\frac{\mu_{hnf}}{\mu_{f}}\right)},\;C_{6}=-\frac{a^{2}b^{2}\frac{dp}{dz}+2a^{2}\left(\frac{\mu_{hnf}}{\mu_{f}}\right)+2b^{2}\left(\frac{\mu_{hnf}}{\mu_{f}}\right)}{2\left(a^{2}+b^{2}\right)\left(\frac{\mu_{hnf}}{\mu_{f}}\right)}, \end{array}$$

Inserting the values of above constants in Equation (22), we have

(23) $$w\left(x,y\right)=-1+\frac{\frac{dp}{dz}\left(\frac{x^{2}}{a^{2}}+\frac{y^{2}}{b^{2}}-1\right)a^{2}b^{2}}{2\left(a^{2}+b^{2}\right)\left(\frac{\mu_{hnf}}{\mu_{f}}\right)},$$

The integral of Equation (23) over the cross-sectional area of this elliptic duct, provides the non-dimensional flow rate given as

(24) $$q\left(z\right)=-ab\pi -\frac{a^{3}b^{3}\frac{dp}{dz}\pi }{4\left(a^{2}+b^{2}\right)\left(\frac{\mu_{hnf}}{\mu_{f}}\right)},$$

where $q\left(z\right)=Q-\int_{0}^{1}abdz,$ and from Equation (24), we have

(25) $$\frac{dp}{dz}=-\frac{4\left(a^{2}+b^{2}\right)\left(-\int_{0}^{1}abdz+ab\pi +Q\right)\left(\frac{\mu_{hnf}}{\mu_{f}}\right)}{a^{3}b^{3}\pi },$$

Finally, the pressure rise is defined as

(26) $$\Delta P=\overset{1}{\underset{0}{\int }}\frac{\partial p}{\partial z}dz,$$

By following a similar procedure for temperature solution, we have

(27) $$\theta \left(x,y\right)=\frac{-a^{2}b^{2}B_{r}{\left(\frac{dp}{dz}\right)}^{2}\left(\frac{x^{2}}{a^{2}}+\frac{y^{2}}{b^{2}}-1\right)\left[b^{6}x^{2}+a^{2}b^{4}\left(b^{2}+6x^{2}-y^{2}\right)+a^{6}\left(b^{2}+y^{2}\right)+a^{4}b^{2}\left(4b^{2}-x^{2}+6y^{2}\right)\right]}{12{\left(a^{2}+b^{2}\right)}^{2}\left(a^{4}+6a^{2}b^{2}+b^{4}\right)\left(\frac{k_{hnf}}{k_{f}}\right)\left(\frac{\mu_{hnf}}{\mu_{f}}\right)}$$

## 5. Results and Discussion

**Figure 2:**
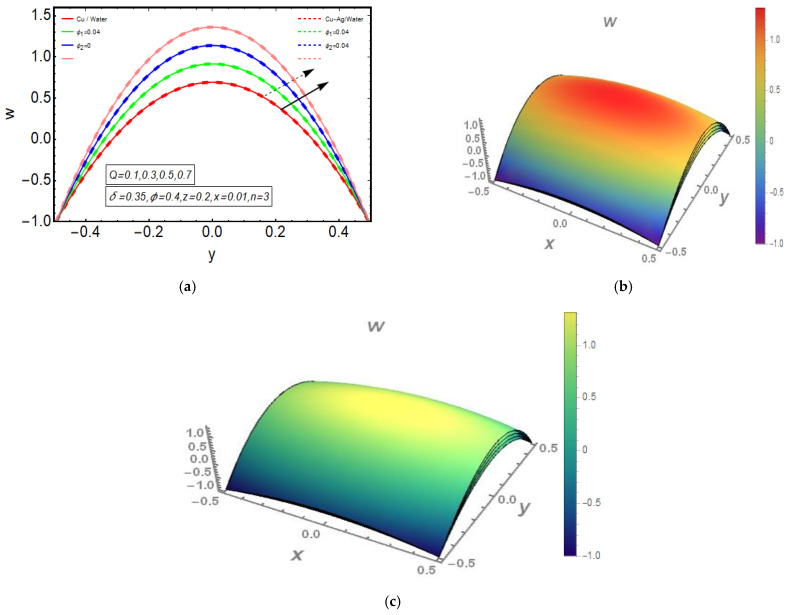
(**a**) 2D-velocity plot for Q. (**b**) 3D-velocity (phase flow model) plot for Q. (**c**) 3D-velocity (hybrid model) plot for Q.

**Figure 3:**
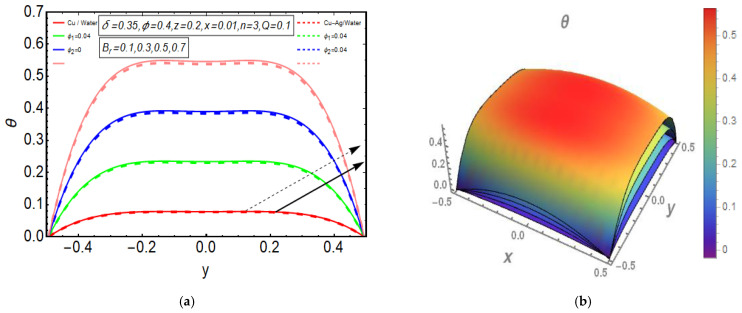

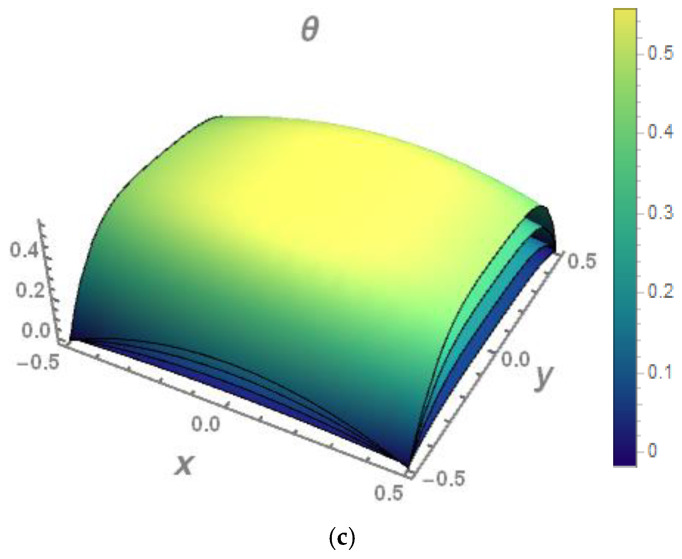
(**a**) 2D-temperature plot for $B_{r}$. (**b**) 3D-temperature (phase flow model) plot for $B_{r}$. (**c**) 3D-temperature (hybrid model) plot for $B_{r}$.

**Figure 4:**
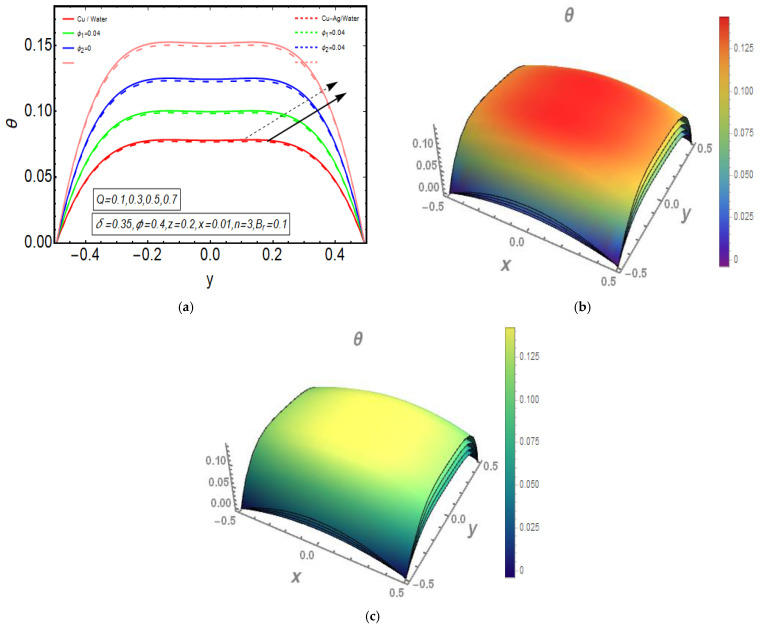
(**a**) 2D-temperature plot for Q. (**b**) 3D-temperature (phase flow model) plot for Q. (**c**) 3D-temperature (hybrid model) plot for Q.

**Figure 5:**
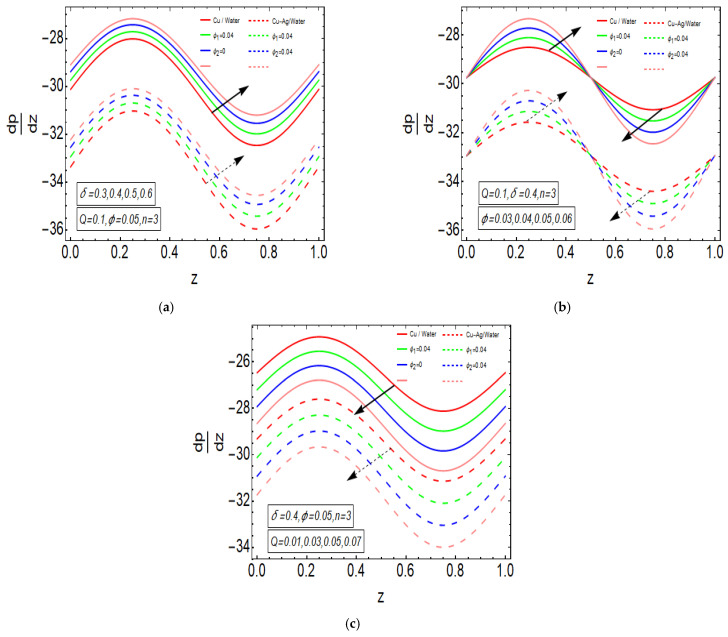
(**a**) $\frac{dp}{dz}$ plot against z−axis for δ. (**b**) $\frac{dp}{dz}$ plot against z−axis for ϕ. (**c**) $\frac{dp}{dz}$ plot against z−axis for Q.

**Figure 6:**
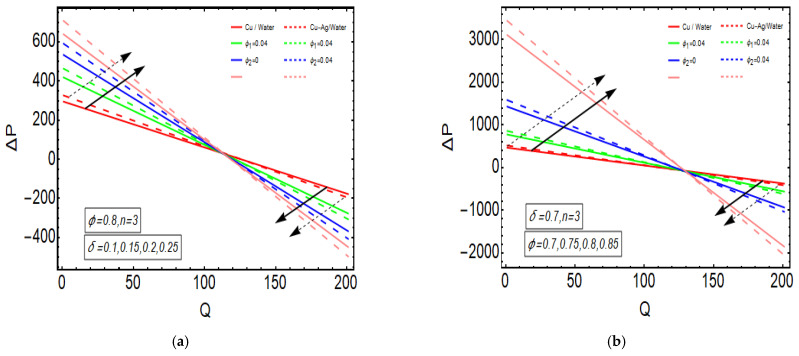
(**a**) ΔP plot against Q for δ. (**b**) ΔP plot against Q for ϕ.

**Figure 7:**
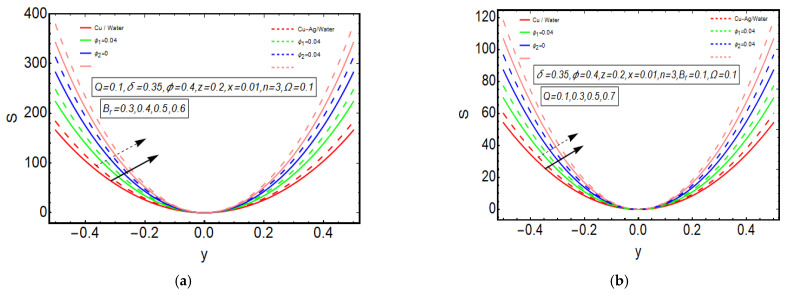
(**a**) Entropy plot for $B_{r}$. (**b**) Entropy plot for Q.

**Figure 8:**
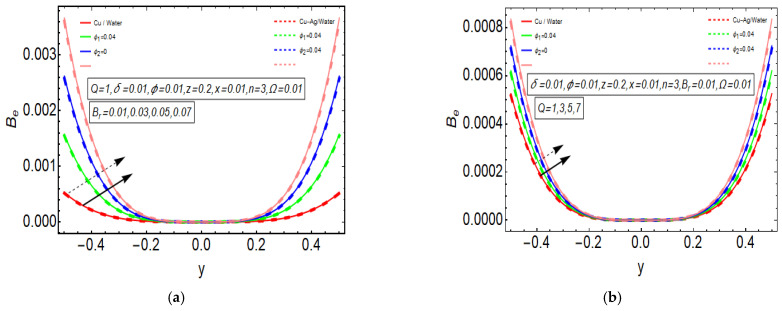
(**a**) $B_{e}$ plot for $$B_{r}$$. (**b**) $B_{e}$ plot for Q.

**Figure 9:**
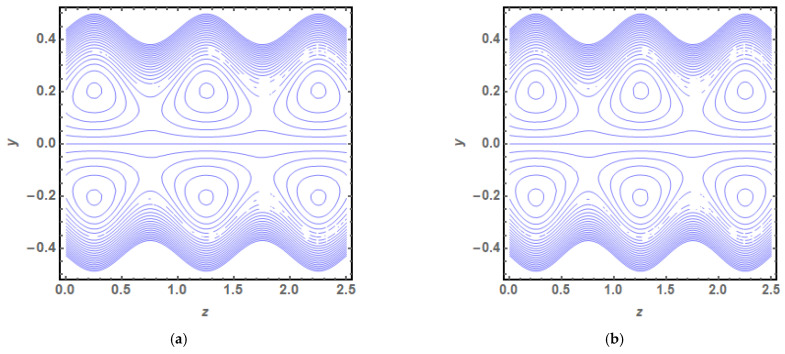

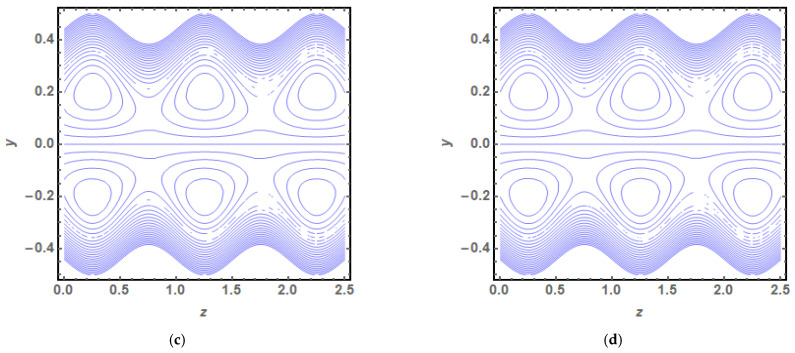
(**a**) Streamline plot (phase flow model) for Q=0.01. (**b**) Streamline plot (phase flow model) for Q=0.02. (**c**) Streamline plot (phase flow model) for Q=0.03. (**d**) Streamline plot (phase flow model) for Q=0.04.

**Figure 10:**
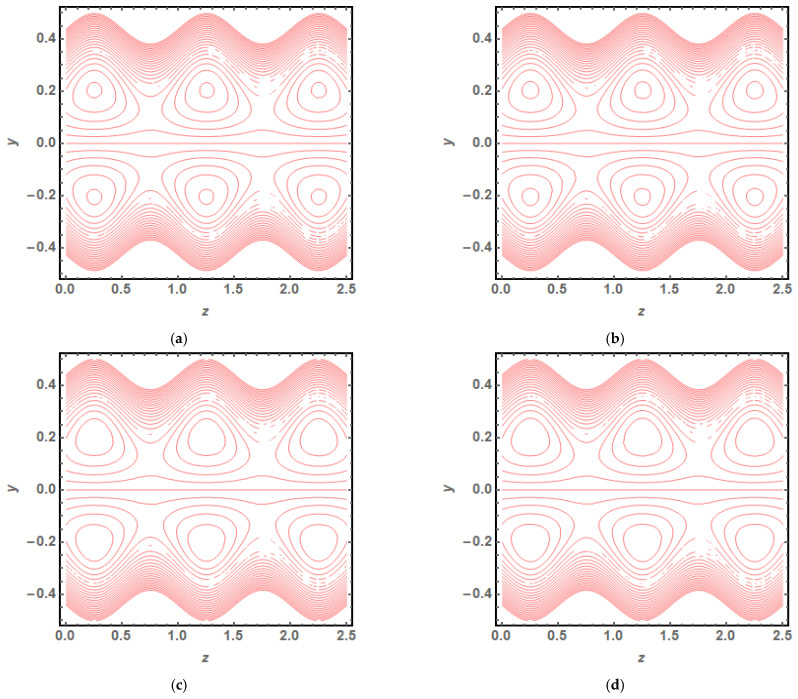
(**a**) Streamline plot (hybrid model) for Q=0.01. (**b**) Streamline plot (hybrid model) for Q=0.02. (**c**) Streamline plot (hybrid model) for Q=0.03. (**d**) Streamline plot (hybrid model) for Q=0.04.

The above exact solution segment discloses an explicit technique that provides exact mathematical outcomes for velocity, temperature, flow rate and pressure gradient. We have also considered the entropy analysis in detail. Presented next is the graphical analysis of these mathematical outcomes that completely verifies the mathematical results. The graphical outcomes disclose a combine analysis for the hybrid nanofluid model (Cu-Ag/water) and phase flow model (Cu/water). In the case of the hybrid model, we have used a four percent concentration for both Cu and Ag with water as a base fluid, while in the case of the phase flow model, only four percent concentration of Cu is used with the base fluid water. The combined graphical results are provided in just a 2D-plot of the graphical outcomes, while we have provided separate graphical solutions as 3D-plots for both the hybrid and phase flow models. Figure 2 reports the graphical solution of velocity for varying values of Q. In Figure 2a, we can see that velocity is increasing at exactly the same rate for both nanofluid models, with increasing Q. Figure 2b presents the 3D-plot of the phase flow model for increasing Q, while Figure 2c discloses the 3D-plot of the hybrid model for increasing Q. A perfectly evolved, parabolic velocity profile also with axial symmetry is observed. The nanofluid’s concentration plays a key role in the enhancement of thermal conductivity of fluid that has its importance in many practical engineering applications. The graphical solutions highlight the results for both phase flow and hybrid models of nanofluids and their effects on various physical parameters. Figure 3 presents the graphical plot of temperature profile for varying values of $B_{r}$. Figure 3a shows that temperature is an increasing function of $B_{r}$ for both of the considered nanofluid models. A slightly rapid increment in temperature is noted for the phase flow model as compared to the hybrid model with increasing $B_{r}$. Figure 3b provides the 3D-plot of the phase flow model for incrementing $B_{r}$, while Figure 3c discloses the 3D-plot of the hybrid model for temperature profile with increasing $B_{r}$. The temperature profile depicts the axially symmetric flow behaviour. Figure 4 demonstrates the effect of Q on the temperature profile and it is observed in Figure 4a that temperature is also an increasing function of Q, since both cases of nanofluid model disclose an increment in temperature for increasing Q. Again, a slightly quicker increase in the temperature is noted for the phase flow model when compared with the hybrid model. Figure 4b discloses the 3D-plot of the phase flow model for increasing Q, while Figure 4c represents the 3D-plot of the hybrid model for temperature profile with incrementing Q. In the core region of the duct, the temperature is notably higher when compared to the boundaries. Figure 5a–c provides the graphical solutions $\frac{dp}{dz}$ plot against z−axis. Figure 5a shows that $\frac{dp}{dz}$ increases for both nanofluid models with increasing δ. A high value of $\frac{dp}{dz}$ is achieved for the phase flow model as compared to the hybrid one. Figure 5b reveals that $\frac{dp}{dz}$ gains high value in both nanofluid models with an expanding peristaltic wave, while the value declines with a relaxing peristaltic wave for incrementing ϕ. Again, the values of $\frac{dp}{dz}$ are higher for the phase flow model as compared to the hybrid model. Figure 5c shows a decline in the value of $\frac{dp}{dz}$ for both nanofluid models with increasing Q. The comparative value of $\frac{dp}{dz}$ is higher for the phase flow model than the hybrid model. Figure 6a,b present the graphical assessment of ΔP plot against Q. Figure 6a reveals that ΔP is an increasing function of δ in the region ΔP>0, while it is a decreasing function of δ in the region ΔP<0 for both nanofluid models. Furthermore, Figure 6b discloses that ΔP gains higher values with increasing ϕ for the region ΔP>0, whereas the value of ΔP declines for increasing ϕ in the region ΔP<0. The entropy analysis S is conveyed graphically through Figure 7a,b. Figure 7a shows the effect of $B_{r}$ on S. The value of S is increasing for both nanofluid models with increasing $B_{r}$. Figure 7b depicts the effect of Q on S and it reveals that S is an increasing function of Q for both of the considered nanofluid models. Higher values of entropy S are noted for the hybrid nanofluid flow as compared to the phase flow. It is disclosed from entropy solutions that the hybrid model of nanofluid causes a higher level of disorder when compared to the phase flow model. Moreover, it is observed that entropy has the lowest values (almost zero) in the core region of the duct due to a fully developed and smooth flow profile in the centre, while entropy S has maximum values near the boundaries due to the sinusoidal fluctuations of walls. Any possible disorder happens due to the sinusoidal wave motion of walls and, therefore, entropy is at its maximum value near boundaries. However, a fully developed flow profile is noted at the centre of the duct so entropy is also at its minimum value at the centre. Figure 8a,b represent the graphical solution of $B_{e}$ for increasing $B_{r}$ and Q, respectively. Figure 8a shows that the value of $B_{e}$ is increasing at the same rate for both nanofluid models with incrementing $B_{r}$. Figure 8b reveals an increase in the value of $B_{e}$ with exactly the same ratio for both nanofluid models with increasing Q. The value of $B_{e}$ approaches zero in the core region of the duct, as entropy is also approaching zero in the core region of the duct. Figure 9a–d provide the streamline plots of the phase flow model for increasing Q. An increment is observed in the trapping phenomenon for increasing Q. Figure 10a–d disclose the streamline plots of the hybrid nanofluid model for increasing Q. Again, a slightly increasing trapping phenomenon is noted for increasing Q.

## 6. Conclusions

The mathematical analysis of peristaltic flow of hybrid nanofluid inside an elliptic duct is presented in this study. Entropy analysis is also incorporated in detail. This is a basic benchmark study that will further develop a key understanding in this area of research. The key outcomes of this mathematical study are narrated as follows:

- A completely evolved, parabolic velocity profile also having axial symmetry is noted.
- A slightly rapid increment in temperature is noted for the phase flow model as compared to the hybrid one with increasing $B_{r}$ and Q.
- The minimum value of disorder in the central region indicates a fully developed flow while the disorder near the walls is due to the sinusoidal fluctuation of boundaries.
- A high value of $\frac{dp}{dz}$ is achieved for the phase flow model as compared to the hybrid model for all the dimensionless parameters involved in this study.
- Higher values of entropy S are noted for the hybrid nanofluid flow as compared to the phase flow.
- The hybrid model of nanofluid is causing a higher level of disorder when compared to the phase flow, as revealed by the entropy solutions.
- It is observed that entropy has its lowest values (almost zero) in the core region of the duct due to a fully developed and smooth flow profile in the centre, while entropy S has its maximum values near boundaries due to the sinusoidal fluctuations of walls.

## Figures and Tables

**Table 1:** Thermophysical properties of base fluid and nano-particles [15].

Physical Parameters	Base Fluid	Nanoparticles	
	(Water)	$\mathrm{Cu}\;(s_{1})$	$\mathrm{Ag}\;(s_{2})$
$C_{p}\left(\frac{J}{kg.K}\right)$	4179	385	235
$k\left(\frac{W}{mK}\right)$	0.613	401	429
$\rho \left(\frac{kg}{m^{3}}\right)$	997.1	8933	10,500

**Table 2:** Hybrid Nanofluid Model [15].

Properties	Nanofluid
Density	$\rho_{hnf}=\left[\left(1-\phi_{2}\right)\left\{\left(1-\phi_{1}\right)\rho_{f}+\phi_{1}\rho_{s1}\right\}\right]+\phi_{2}\rho_{s2},$
Viscosity	$\mu_{hnf}=\frac{\mu_{f}}{{\left(1-\phi_{1}\right)}^{2.5}{\left(1-\phi_{2}\right)}^{2.5}},$
Thermal Conductivity	$\frac{k_{hnf}}{k_{bf}}=\frac{k_{s2}+\left(n-1\right)k_{bf}-\left(n-1\right)\phi_{2}\left(k_{bf}-k_{s2}\right)}{k_{s2}+\left(n-1\right)k_{bf}+\phi_{2}\left(k_{bf}-k_{s2}\right)}$ $\frac{k_{bf}}{k_{f}}=\frac{k_{s1}+\left(n-1\right)k_{f}-\left(n-1\right)\phi_{1}\left(k_{f}-k_{s1}\right)}{k_{s1}+\left(n-1\right)k_{f}+\phi_{1}\left(k_{f}-k_{s1}\right)}$
Heat Capacity	${\left(\rho C_{p}\right)}_{hnf}=\left[\left(1-\phi_{2}\right)\left\{\left(1-\phi_{1}\right){\left(\rho C_{p}\right)}_{f}+\phi_{1}{\left(\rho C_{p}\right)}_{s1}\right\}\right]+\phi_{2}{\left(\rho C_{p}\right)}_{s2},$

## Nomenclature

$\left(\bar{X},\;\bar{Y},\;\bar{Z}\right)$	Coordinate system
$\left(\bar{U},\;\bar{V},\;\bar{W}\right)$	Velocity components
δ	Aspect ratio
$\phi_{1}$	Concentration of copper
$\phi_{2}$	Concentration of silver
$\rho (\frac{\mathrm{kg}}{m^{3}})$	Density
Ω	Dimensionless temperature ratio
ϕ	Occlusion
$\mu ({\mathrm{Nsm}}^{-2})$	Viscosity
λ (m)	Wavelength
$B_{r}$	Brinkman number
$a_{0},b_{0}$	Ellipse half axes $\left(b_{0}<a_{0}\right)$
$c\left({\mathrm{ms}}^{-1}\right)$	Wave velocity
d(m)	Wave amplitude
e	Ellipse eccentricity
$k\left(\frac{W}{\mathrm{mK}}\right)$	Thermal conductivity
S	Dimensionless entropy
${\bar{T}}_{w}\left(K\right)$	Tube’s wall temperature
$D_{h}\left(m\right)$	Hydraulic diameter of ellipse
$C_{p}\left(\frac{J}{\mathrm{kg}.K}\right)$	Heat capacity
$h_{nf}$	Hybrid nanofluid
${\bar{T}}_{b}\;\left(K\right)$	Bulk temperature
